# Electrophysiological Responses and Reproductive Behavior of Fall Webworm Moths (*Hyphantria cunea* Drury) are Influenced by Volatile Compounds from Its Mulberry Host (*Morus alba* L.)

**DOI:** 10.3390/insects7020019

**Published:** 2016-05-03

**Authors:** Rui Tang, Feng Zhang, Zhong-Ning Zhang

**Affiliations:** 1 State Key Laboratory of Integrated Management of Pest Insects and Rodents, Institute of Zoology, Chinese Academy of Sciences, 1-5 West Beichen Road, Beijing 100101, China; zhangzn@ioz.ac.cn; 2 CAB International East Asia Regional Center, Chinese Academy of Agricultural Sciences, 12 Zhongguancun Nandajie, Beijing 100081, China; f.zhang@cabi.org; 3 MoA-CABI Joint Laboratory for Bio-safety, Institute of Plant Protection, Chinese Academy of Agricultural Sciences, 2 West Yuan-ming-yuan Road, Beijing 100193, China

**Keywords:** mulberry moth, HIPVs, electroantennogram, mating, oviposition

## Abstract

*Hyphantria cunea* (Drury) is an invasive pest of *Morus alba* L. in China. β-ocimene and *cis*-2-penten-1-ol among eleven electro-physiologically active leaf volatiles from *M. alba* have been reported to influence captures of *Hyphantria cunea* moths when added into sex pheromone traps. This study further investigated influences of volatile types and their dosages on the electro-physiological responses in the antennae of male and female moths, as well as on mating and oviposition behaviors. Females were, regardless of dosages, more sensitive to β-ocimene and *cis*-2-penten-1-ol in electro-physiological response tests than males. For males, a dose response was detected, *i.e.*, a dosage of 10 μg and 100 μg of either chemical stimulated higher electric response in their antennae than 1 μg. Moth pairs either exposed respectively to a herbivore-induced *M. alba* volatile blend (HIPV), to a mechanically-damaged *M. alba* volatile blend (MDV), to β-ocimene, to *cis*-2-penten-1-ol, or to pentane as a control showed that pairs exposed to β-ocimene most likely mated, followed by HIPV blends and least by the other volatiles or the control. In contrast, β-ocimene induced about 70% of the female oviposition behaviors and was nearly 4.5 times the oviposition rate than *cis*-2-penten-1-ol and 2 times than the control. However, none of the chemicals had any effect on the 48 h fecundity or on egg sizes. In conclusion, β-ocimene from mulberry plants alone could promote mating and oviposition in *H. cunea* at a dosage of 1 mg. The results indicate that reproductive behaviors of *H. cunea* moths can be enhanced through HIPV blends and β-ocimene induced by feeding of larvae. This contra phenomenon has revealed a different ecology in this moth during colonizing China as local pests would commonly be repelled by herbivore induced chemicals. These chemicals can be used for the development of biological control approaches such as being used together with sex pheromone traps.

## 1. Introduction

The fall webworm, *Hyphantria cunea* Drury (Lepidoptera: Arctiidae), is native to North America but spread into central Europe and eastern Asia in the 1940s [1,2]. In 1979, this insect was first recorded in China in Liaoning Province, and it has continued to spread through much of the country [3]. *Hyphantria cunea* has caused extensive damage during its 30-year invasion because it feeds on 175 species of host plants [4]. However, it prefers to eat mulberry (*Morus alba* L., Moraceae), rock maple (*Acer saccharum* Marshall, Sapindaceae), poplar (*Populus tremula* L., Salicaceae), and ailanthus (*Ailanthus altissima* (Mill.) Swingle, Simaroubaceae) in China [3]. Economically-important mulberry plants are mostly heavily damaged because of their long cultivation history over 4000 years, board area across the country and high densities in China [5].

Although methods to control this moth in China using sex pheromones and biological control via parasitoid wasps have been systematically studied [3,4], research has focused on its molecular biology, development, and genetics [6,7,8]. However, studies on roles of plant volatiles in *H. cunea* have been rare, and nearly all have addressed host location theories [9,10].

Herbivore-induced plant volatiles (HIPVs) function as signal chemicals for insects in nature and are involved in plant defensive functions in various ways [11,12]. For example, HIPVs can attract natural enemies of plant pests [13,14]. HIPVs may also affect attraction of sex pheromones in some insects and affect the production and release of methyl-branched hydrocarbons [15,16,17,18,19,20,21].

In previous studies, some volatile compounds from mulberry leaves have been identified [22,23,24,25,26], and the electrophysiological effects on *H. cunea* of some botanical volatile compounds, such as green leaf volatile relevant aldehydes, alcohols, esters and so on have been studied [26]. Mechanically damaged mulberry leaves emitted volatiles with only proportion changed, however, cutting would not induce the emission of unique volatile compounds compared with intact leaves. When compared with HIPVs, *cis*-2-penten-1-ol was only detected in MDVs while β-ocimene was only detected in HIPVs. In addition, we previously showed that they are the only two mulberry compounds so far found to influence *H. cunea* trapping in the field [27]. However, little is known about how mulberry HIPVs affect *H. cunea*’s reproductive behavior.

Mating and oviposition in some insects can be interfered by plant volatile compounds as antennae sensitivities to pheromone components could be influenced [28,29,30]. To test whether the reproduction of *H. cunea* could be similarly affected, we used mulberry HIPVs, MDVs, β-ocimene, and *cis*-2-penten-1-ol for testing their performances towards moth pairs. Our aims were to examine the potential effects of HIPVs, MDVs, β-ocimene and *cis*-2-penten-1-ol on the reproduction of *H. cunea* and to compare volatile chemical blends with β-ocimene and *cis*-2-penten-1-ol in order to reveal their functions and potential usages toward controlling of this invasive pest.

## 2. Materials and Methods

### 2.1. Origin and Handling of Hyphantria cunea

Insects were provided as pupae by the Chinese Academy of Forestry (Beijing, China), which established a laboratory population of *H. cunea* using wild moths collected in Qinghuangdao, Hebei Province, China in 2008. Pupae were firstly separated by sexes and then kept at 25 ± 1 °C under a 16 h light/8 h dark photoperiod in climate chambers before emergence. Emerged adult moths were kept at the same temperature and humidity with 10% honey water provided. Third-instar larvae were starved for 1day before the experiment of head space absorption.

### 2.2. Tested Volatiles

Chemicals used in the experiments are listed in Table 1. All glassware was carefully cleaned, washed with acetone, and heated at 200–230 °C overnight before use. The absorbent Super Q GC Packed Column (80/100, Alltech, Deerfield, IL, USA) used to collect volatiles in air entrainment experiments was washed with acetone, tested for purity by gas chromatography (GC), and then conditioned by heating overnight in a stream of nitrogen at 100 °C.

We conducted two mulberry treatments, each with five samplings. (1) Mechanically-damaged leaf volatile compounds: Volatiles were collected from mechanically-damaged leaves. A total 100 leaves were chosen and 25 leaves were randomly cut in half. Then the chosen mulberry leaves were encased in oven bags (482 mm × 596 mm, Reynolds, Richmond, VA, USA) on the trees. (2) For collecting herbivore-induced leaf volatile compounds (HIPVs), ten *H. cunea* larvae were placed on mulberry leaves encased in oven bags, and allowed to feed for 1 h. Then larvae and their excrement were removed.

For all treatments, air was collected via an air sampler (QC-1, Beijing Municipal Institute of Labor Protection, Beijing, China) and 0.3 g of Super Q adsorbent connected to the oven bags. Air flowing into the bags was purified by an activated carbon sphere on the bag inlet and air flows at 50 mL/min. Volatile samples were collected for 24 h before elution with 2 mL of solvent (70% pentane, 30% diethyl ether) and 72 L air was captured via absorbent in total. Eluted samples were frozen at −20 °C prior to the moth experiments.

### 2.3. Assessing Effects of Morus alba Volatiles β-ocimene and cis-2-penten-1-ol on EAG Responses of Hyphantria cunea

The electroantennogram (EAG) method was adopted from [31] to test differences of male and female responses to β-ocimene and *cis*-2-penten-1-ol. Each antenna was prepared by cutting both extremes and immediately mounted between two Ag/AgCl electrodes filled with Kaissling saline (glucose 354 mmol/L, KCl 6.4 mmol/L, KH_2_PO_4_ 20 mmol/L, MgCl_2_ 12 mmol/L, CaCl_2_ 1 mmol/L, NaCl 12 mmol/L, KOH 9.6 mmol/L, pH 6.5). The electrode at the distal end of the antenna was connected via an interface box to a signal acquisition interface board (IDAC; Syntech, Kirchzarten, Germany) connected to a computer. EAG signals were recorded by a computer using AutoSpike software (Syntech, Irvine, CA, USA).

Each of these compounds was purchased in pure form and diluted to 10 mg/mL with solvent (70% pentane and 30% diethyl ether). A gas control unit (Syntech CS-05) gave an air flow rate of 30 mL/s with the gas outlet facing the antenna and 1 cm distance from it. For each solution, 10 µL was dropped on a 0.5 × 5 cm filter paper in a Pasteur pipette. The Pasteur tube was placed between the gas control unit and the gas outlet.

Antennae from both sexes of *H. cunea* were stimulated for 0.1 s with an interval time of 1 min. Nonanal was used as a standard compound and solvent was used as the control. Tests were run in the following sequence: control, nonanal, sample, control, nonanal. Each antenna was tested twice and each compound was tested with 10 different antennae per gender. Electroantennogram data was adjusted to eliminate the interference of solvent and environment using the formula:

*Electroantennogram value = R_C_ − (R_C−1_ − R_C+1_)/2 *

where R_C_ is the raw electroantennogram value of the sample, R_C-1_ is the value of the control before the sample, and R_C+1_ is the value of control after the sample [32].

The adjusted EAG value was expressed as a specific value by dividing by the standard compound value. Mean values were tested using one-way ANOVA, and means were compared with Tukey HSD at *p* = 0.05. Each compound’s mean difference between male and female moths was tested using General Linear Model.

Chemical concentrations for dose responses were: 0.1, 1, 10 and 100 mg/mL. Dosages of each stimulation were 10 μL × (0.1, 1, 10 and 100 mg/mL) = 1, 10, 100 and 1000 μg. Test and data analysis methods were same as above, and at least 6 antennae were tested for each dosage of each chemical compound.

### 2.4. Assessing Effects of Morus alba HIPV, MDV, β-ocimene and cis-2-penten-1-ol on Hyphantria cunea Mating and Oviposition

Newly-emerged moths were kept at 25 ± 1 °C under a 16 h light/8 h dark photoperiod in environmental chambers, and 1–3 day old adult moths were used in experiments.

Gray rubber septa (The West Company, Phoenixville, PA, USA) loaded with 1000 μg (100 μL at 10 mg/mL) of either β-ocimene or *cis*-2-penten-1-ol were used in these experiments for mating and ovipostion tests. This dosage was chosen according to previous electroantennogram results [26]. HIPV and MDV samples were added 100 μL per septum. Concentrations of total volatile compounds were at an average of 10.96 μg/μL. The average of total mass of volatile organic compounds captured by each absorbent was 21.92 mg by calculating with external standard method [27]. The solvent alone was used as control.

A pair (one male, one female) of virgin moths was placed in a beaker (2000 mL, d = 130 mm, h = 185 mm) together with one loaded septum. Experiments began at the 6th h of the dark phase to cover calling peaks of female moths [3], and the number of pairs that mated within 2 h was observed and counted.

Mating rate was defined as the proportion of pairs who conducted mating behaviors within every group (five independent pairs, so the value was intervals of 0, 0.2, 0.4, 0.6, 0.8 and 1.0; this set-up was to reduce variation among replicates since 1 pair per sample size would lead to an interval value between 0 and 1, which would have resulted in larger standard deviation). Oviposition rate was similarly defined as the proportion of oviposition observed within every group.

Egg numbers were counted after 48 h in every pair with oviposition observed, and average egg sizes were calculated by random measurement of egg masses. The effects of the compounds on moth behavior were analyzed using one-way ANOVA. Tukey HSD multiple-comparison test was used to compare mean differences between groups at *P* = 0.05. All analyses were performed in SPSS 20.0 (IBM, Chicago, IL, USA).

## 3. Results

### 3.1. Effects of Morus alba Volatiles β-ocimene and cis-2-penten-1-ol on EAG Responses of Hyphantria cunea

**Figure 1:**
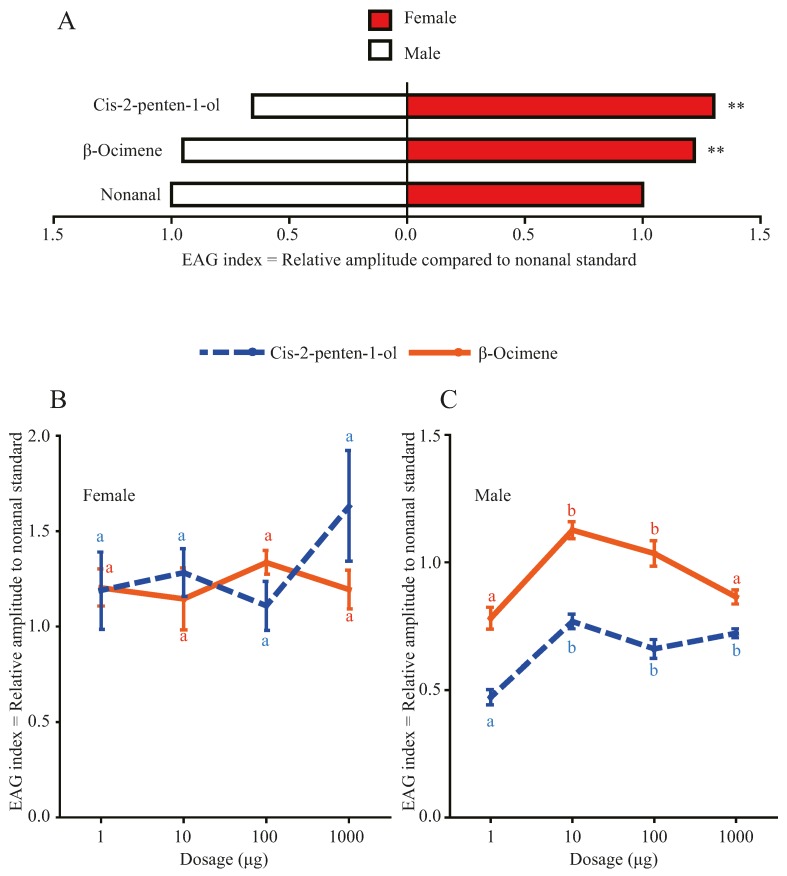
Comparison of electrical response intensities in antennae of male and female *Hyphantria cunea* moths towards the mulberry volatiles β-ocimene and *cis*-2-penten-1-ol: (**A**) averaged across dosages with asterisks indicating significant differences of electric responses level between sexes according to GLM at *p* < 0.05, n = 53; (**B**) of females per dosages, n = 7 and (**C**) of males per dosages, n = 6; with letters indicating significant differences between the two volatiles according to Tukey HSD after ANOVA at *p* < 0.05; Error bars = SEM, EAG = electroantennogram.

Electroantennogram analyses showed that female moths had higher electro-physiological responses to the tested mulberry volatiles than male moths (Figure 1A). Female antennae responded with significantly higher amplitudes to both β-ocimene (GLM, F_1,53_ = 20.5, *p* < 0.001, Figure 1A) and *cis*-2-penten-1-ol (GLM, F_1,54_ = 49.3, *p* < 0.001) than males. No significant differences were observed in females among the four tested dosages of either β-ocimene (ANOVA, F_3,19_ = 0.53, *p* = 0.67, Figure 1B) or *cis*-2-penten-1-ol (ANOVA, F_3,20_ = 1.32, *p* = 0.294). Males, responded with higher intensities to 10 μg and 100 μg β-ocimene than to 1 μg and 1000 μg (ANOVA, F_3,28_ = 15.78, *p* < 0.001; all Tukey HSD tests *p* < 0.05). The three dosages of 10 μg, 100 μg and 1000 μg *cis*-2-penten-1-ol elicited comparable; but significantly higher responses in the antennae of male moths than did 1 μg (ANOVA, F_3,28_ = 19.89, *p* < 0.001; all Tukey HSD tests *p* < 0.05).

### 3.2. Effects of Morus alba HIPV, MDV, β-ocimene and cis-2-penten-1-ol on Hyphantria cunea Mating and Oviposition

**Figure 2:**
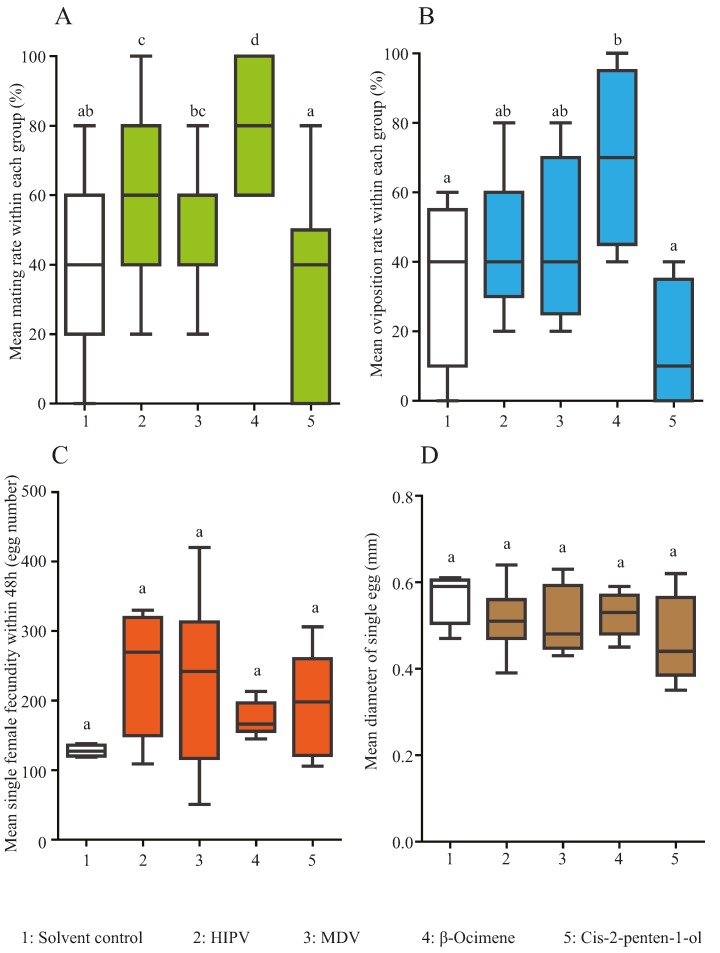
Comparison of influences of different volatiles on (**A**) mating rates calculated within each group (5 pairs, n = 74); (**B**) on oviposition rates calculated within each group (5 pairs, n= 16); (**C**) on average single female fecundity within 48 h (n = 26) or (**D**) on average eggs sizes (n =38). 1: solvent control; 2: HIPV = herbivore-induced mulberry plant volatile blend; 3: MDV = mechanical damage-induced mulberry volatile blend; 4: β-ocimene standard chemical; 5: *cis*-2-penten-1-ol standard chemical. Error bars = SEM; Different letters on bars indicate significant differences according to ANOVA and Tukey HSD at *p* < 0.05.

Figure 2A,B summarize the effects of different volatile compounds on *H. cunea* mating and oviposition behaviors. The average mating rates calculated for each compounds were 38% ± 7% for solvent control, 58% ± 5% for HIPVs, 46% ± 4% for MDVs, 78% ± 5% for β-ocimene and 31% ± 7% for *cis*-2-penten-1-ol. The average oviposition rates calculated for each compounds were 35% ± 13% for solvent control, 44% ± 10% for HIPVs, 45% ± 13% for MDVs, 70% ± 13% for β-ocimene and 15% ± 10% for *cis*-2-penten-1-ol.

Significant differences of mating rates were found among the five treatments (ANOVA, F_4,74_ = 9.5, *p* < 0.001). In detail, β-ocimene led to the highest mating rates of moth pairs, and about 2 times of those in control treatment (Tukey HSD, *p* < 0.0001). HIPVs also significantly increased mating rates by almost 50% compared to control (Tukey HSD, *p* = 0.01). In contrast, moths exposed either to the MDV (Tukey HSD, *p* = 0.32) blend or to *cis*-2-penten-1-ol (Tukey HSD, *p* = 0.36) did not change mating rates compared with solvent control. MDV treatment resulted in a significantly higher mating rate than did the *cis*-2-penten-1-ol one (Tukey HSD, *p* = 0.047, Figure 2A).

Significant differences among oviposition rates were observed among treatments (ANOVA and Tukey HSD, F_4,16_ = 2.86, *p* = 0.031, Figure 2B). In detail, exposure to β-ocimene resulted in the highest average oviposition rates, this is, about 4.5 times compared to *cis*-2-penten-1-ol and 2 times to the solvent (Tukey HSD, *p* = 0.031). None of the other treatments differed significantly from each other in their effects on oviposition rates.

The average egg numbers laid by a single female within 48 h calculated for each compound were 128 ± 4 eggs for solvent control, 243 ± 37 eggs for HIPVs, 240 ± 47 eggs for MDVs, 174 ± 8 eggs for β-ocimene and 192 ± 35 eggs for *cis*-2-penten-1-ol. The average egg diameters calculated for each compound were 0.56 ± 0.03 mm for solvent control, 0.51 ± 0.02 mm for HIPVs, 0.51 ± 0.02 mm for MDVs, 0.53 ± 0.01 mm for β-ocimene and 0.47 ± 0.05 mm for *cis*-2-penten-1-ol.

No significant differences were observed among chemical effects on female 48 h fecundity (GLM, F_4,26_ = 1.57, *P* = 0.21, Figure 2C) or on egg sizes (GLM, F_4,38_ = 1.43, *P* = 0.24, Figure 2D).

## 4. Discussion

Changes in chemical volatile profiles have been repeatedly reported throughout many host plants when they are fed on by pest insects [28] or mechanically damaged. Oral secretion-induced plant volatile changes have also been reported having essential functions towards female moth oviposition [30]. For example, β-ocimene and *cis*-2-penten-1-ol have been reported as volatiles from mulberry leaves and their existences and emission volume changed dramatically during feeding by *H. cunea* larvae [27]. These botanical volatile chemical blends showed bioactivities on *H. cunea* adults in laboratory experiments such as electroantennogram tests and in wind tunnel experiments. Furthermore, the two compounds seem to also affect long distance host location of *H. cunea* in the field and were suggested to be synergists for *H. cunea* sex pheromones [25,28]. However, we did not know their short distance functions especially when they are presented together with sex pheromones.

In this study, we found that HIPVs from mulberry could significantly increase the mating rate compared with the solvent control (treatment No. 1). This result implies that mulberry HIPVs may act differently on *H. cunea* than on most regional pests, which are usually repelled by the HIPVs [30,33]. Although we showed an increased mating as a result of HIPV in our study, the oviposition rate was not enhanced, and this might be the result of potential bioactive compounds in the blends of HIPV compounds other than β-ocimene. HIPV blends may have complex effects on moth behaviors because of their multiple constituents, and this complexity can be more variable in the environment than in the lab [27]. The enhancement of *H. cunea* mating by mulberry HIPVs may make this moth more adaptable to this plant than local insect herbivores. In futures studies, we will assess which parts/compounds and dosages may have which effects on their own.

Higher EAG amplitudes stimulated by both chemicals implies that female moths are more sensitive to host volatile compounds than males. In addition, this may result of the female interest to find suitable sites for their offspring. It might be evolutionarily risky for females to prefer chemicals, which are also preferred by their natural enemies. β-Ocimene is a monoterpene, a natural 10-carbon member of the terpenoid family, which are common constituents of floral scents that attract insects including natural enemies or pollinators [34]. Ocimene activates defensive responses in *Arabidopsis thaliana* [35,36] and functions as a volatile brood pheromone involved in social regulation in honeybee colonies [37]. For example, z-ocimene was also reported to have a function in inducing herbivory in a number of plant species [35,38,39,40]. Interestingly, in some of the reported works, β-ocimene was considered neutral for chemical preferences of natural enemies like parasitoids [41]. Therefore, oral secretion induction and attractiveness to this chemical would benefit *H. cunea* since the chemical would not attract their natural enemies. This oviposition preference to herbivore-induced volatiles of *H. cunea* moths might also reveal a potential novel invading model which is different from local pest species. This model has similarities with other invasive pests in China, such as red turpentine beetle *Dendroctonus valens* [42], and this might be a result of a lack in local natural enemies with the same chemical odorant preferences.

Our experiments showed that β-ocimene increased both the mating and oviposition rates in *H. cunea*. This implies that HIPVs and β-ocimene may affect the moths when presented together with sex pheromone compounds because sex pheromones were emitted by females during the calling when we conducted the experiments in dark phase. Similar conclusions have been reported that volatile organic compounds could enhance the ability of antennal sensors to better recognize pheromones [15,43]. Thus, β-ocimene could increase the efficiency of sex pheromone traps for *H. cunea* moths [26]. This indicates that β-ocimene may function in both long distance (trapping) and short distance (mating). As HIPVs enhanced mating behaviors in short distance as shown in the lab, but did not affect long distance location of male moths as shown in [26], we suggest that the short distance function of those chemicals is primarily for mating, as also suggested by [44].

It has been reported that host plant odorant volatiles could be employed as insect sex pheromone components and were necessary for some insects to conduct mating and oviposition [28]. However, this was not observed in *H. cunea* since mating behaviors were found also for the solvent control (pentane + ether) treatment within which no VOCs were present. This finding supports previous works which reported that sex pheromone blends of *H. cunea* moths were composed of (9Z,12Z)-octadecadienal, (9Z,12Z,15Z)-octadecatrienal, (3Z,6Z,9S,10R)-9,10-epoxy-3,6-heneicosadiene and (3Z,6Z,9S,10R)-9,10-epoxy-1,3,6-heneicosatriene, none of which was found in mulberry volatiles [3].

*Cis*-2-penten-1-ol was reported to be present in peppermint oil [45], which repels many insects. Moreover, *cis*-2-penten-1-ol has been discovered in tea leaves [46]. Interestingly, tea tree is not a host plant for *H. cunea* [47]. This implies that at least *cis*-2-penten-1-ol would not be preferred by this moth for host location, or like it has been reported, would even repel this moth [27]. In addition, the moth may overcome this problem by changing the volatile chemical profile of host plants during the feeding process. When testing mating and oviposition, *cis*-2-penten-1-ol showed no effect on the moths` behavior compared with solvent control. This indicates that *cis*-2-penten-1-ol is more likely a long distance function compound than β-ocimene, and it acts maybe more on male than on female moths. We also speculate that this compound may affect a metabolic process instead of the olfactory based behavior reflection itself. Influences of metabolic targeting compounds are often expressed in the following generation of insects [48,49]. Therefore, the influences of *cis*-2-penten-1-ol might also occur after a long term exposure and reveal a tradeoff between egg number and egg size [50].

## 5. Conclusions

In conclusion, the tested mulberry compounds, β-ocimene and *cis*-2-penten-1-ol, were not essential for *H. cunea* moths to mate and oviposit. However, mulberry volatile compounds did affect reproduction behaviors in *H. cunea* by increasing the mating rate and oviposition rate. As β-ocimene can also work as synergist with sex pheromone compounds for helping in the location of male moths [26], we suggest that the blend of HIPVs can function both in long distances (e.g., for female or host location) and short distances (mating and oviposition). In addition, β-ocimene was only induced by *H. cunea* larvae feeding on the mulberry host plants, which indicates that these moths prefer to feed and reproduce on previously-damaged plants. This aggregated attacking model has also been reported for explaining the colonization of other invasive species in China, such as red turpentine beetle. Our study has revealed a conflict that invasive *H. cunea* would treat HIPVs differently from local pests as they preferred the herbivore-induced component β-ocimene. However, the olfactory perception behind this phenomenon still needs to be discovered.

## Figures and Tables

**Table 1:** Chemicals used in electro-physiological tests as well as in mating and oviposition bioassays. β-ocimene and *cis*-2-penten-1-ol represent host volatiles, and other chemicals were used as controls, solvents and washing.

Chemical	Type	Test	Tested insects	Purity	Source
Pentane ^1^ & ether ^2^	Solvent	EAG & bioassay	sample size 5 independent pairs, 5 replicates	Analytically pure **	^1^ Shantou Xilong Chemical Factory, Guangdong, China ^2^ Beijing Chemical Factory, Beijing, China
Acetone	Washing	-	-	Analytically pure **	Beijing Chemical Factory, Beijing, China
Nonanal	Standard compound	EAG	All tested antennae	GC pure	Sigma-Aldrich, St. Louis, MO, USA
β-ocimene	Volatile from HIPVs	EAG & bioassay	sample size 5 independent pairs, 5 replicates	95%	Sigma-Aldrich, St. Louis, MO, USA
*cis*-2-penten-1-ol	Volatile from MDVs	EAG & bioassay	sample size 5 independent pairs, 5 replicates	95%	Tokyo Chemical Industry Co., Tokyo, Japan
HIPVs	Volatile from larvae-damaged *Morus alba*	bioassay	sample size 5 independent pairs, 5 replicates		Produced by head space absorption method
MDVs	Volatile from mechanically-damaged *Morus alba*	bioassay	sample size 5 independent pairs, 5 replicates		Produced by head space absorption method

** tested with gas chromatography for confirmation of their purity.
